# Long-term effects of arterial hypertension on the choroid plexus, blood to cerebrospinal fluid barrier and CSF proteins and their involved in certain types of dementia

**DOI:** 10.1186/1743-8454-7-S1-S29

**Published:** 2010-12-15

**Authors:** Agustin Castañeyra-Perdomo, Ibrahim Gonzalez-Marrero, Rafael Castro, Agustin Castañeyra-Ruiz, Hector de Paz-Camona, Leandro Castañeyra-Ruiz, Juan M Gonzalez-Toledo, Emilia M Carmona-Calero

**Affiliations:** 1 Departamento de Anatomía, Facultad de Medicina, Universidad de La Laguna, Tenerife, Spain; 2 Departamento de Biotecnología, Instituto de Investigación y Ciencias de Puerto del Rosario, Fuerteventura, Spain; 3 Departamento de Fisiologia, Facultad de Medicina, Universidad de La Laguna, Tenerife, Spain

## Background

The correlation of hypertension with vascular dementia has long been established and it is becoming increasingly clear that hypertension is also a reversible risk factor in the development of Alzheimer’s dementia (AD). Aging, AD and hypertension are major determinants of cognitive dysfunction which are associated with profound alterations in the structure and function of cerebral blood vessels. The aim of the present work is to analyze cerebrovascular blood flow in the middle cerebral artery (MCA), the integrity of blood to CSF barrier (BCB), choroid plexus (CP) structure and protein secretion as well as cerebrospinal fluid (CSF) protein composition.

## Materials and methods

Choroid plexus (CP), CSF and serum from hypertensive (SHR) and control (WKY) rats of 8, 26 and 52 weeks of age were used. Blood flow was measured by transcranial Doppler sonography on MCA. Brain sections containing CP were processed by immunohistochemistry using antibodies against transthyretin (TTR), caspase-3 (CAS-3), proliferating cell nuclear antigen (PCNA) and immunoglobulin G (IGG). CSF from 26 weeks of age was used by 2D electrophoresis and western-blot.

## Results

CP immunohistochemistry: TTR increased during aging in SHR with respect to WKY. CAS-3 was undetectable at 8 weeks in SHR and at 8 and 26 weeks in WKY, at 26 weeks the SHR showed a slight reaction which was increased at 52 weeks of age, a soft reaction was also observed at 52 weeks in WKY rats, SHR also expressed a significant number of cells marked by CAS-3. PCNA results were similar to CAS-3; IGG was mainly expressed in the basolateral membrane of the CP and in CP cells at 52 weeks of age in SHR. 2D electrophoresis and Western-blot: By 2D, we found differences in the CSF protein profile of the SHR when compared to CSF of the WKY, these variations were similar to variations found by other authors in AD. By Western-blot (WB) the TTR was found to be higher in blood and lower in the CSF of SHR with respect to WKY groups.

## Conclusions

Long-term effects of hypertension cause a degeneration and hypertrophy of the CP producing variations in protein secretion to the CSF that could influence the maintenance of neurons and functions in the brain. Variations in CSF proteins in AD could similarly cause a CP disruption and support the close connection between cerebrovascular risk factors and AD in light of the fact that they could share common pathogenic etiological processes.

